# All‐in‐One Process for Color Tuning and Patterning of Perovskite Quantum Dot Light‐Emitting Diodes

**DOI:** 10.1002/advs.202200073

**Published:** 2022-03-01

**Authors:** Junho Kim, Ki‐Won Seo, SeungJae Lee, Kyungmin Kim, Changjo Kim, Jung‐Yong Lee

**Affiliations:** ^1^ School of Electrical Engineering (EE) Korea Advanced Institute of Science and Technology (KAIST) 291 Daehak‐ro, Yuseong‐gu Daejeon 34141 Republic of Korea

**Keywords:** perovskite colloidal quantum dot light emitting diode, all‐in‐one process, film‐state ligand exchange, color tuning, patterning

## Abstract

Although post‐synthetic anion exchange allows halide perovskite quantum dots to easily change the optical bandgap of materials, additional exchange of shorter ligands is required to use them as active materials in optoelectronic devices. In this study, a novel all‐in‐one process exchanging ligands and halide anions in film‐state for facile color tuning and patterning of cesium lead halide perovskite colloidal quantum dot (PeQD) light‐emitting diodes (LEDs) is proposed. The proposed all‐in‐one process significantly enhances the performances of PeQD LEDs by passivating the PeQD with shorter ligands. In addition, the all‐in‐one process is repeated more stably in the film state. Red, green, and blue LEDs with extremely narrow emission spectra using cesium lead bromide PeQDs and appropriate butylammonium halide solutions are fabricated. Furthermore, the proposed all‐in‐one process in film‐state facilitated rapid color change in localized areas, thereby aiding in realizing fine patterns of narrow widths (300 µm) using simple contact masks. Consequently, various paint‐over red/green/blue patterns in PeQD LEDs by applying halide solutions additively are fabricated.

## Graphical abstract

A novel all‐in‐one process with simultaneous ligand and anion exchange for cesium lead halide perovskite quantum dot (PeQD) light‐emitting diodes (LEDs) is proposed. Through the all‐in‐one process, it can enhance the PeQD LED performance and easily convert the emitting light color. Furthermore, the various patterned LEDs without direct exposure of ultraviolet ray or electron‐beam to PeQD layer can be realized.

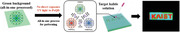

## 1 Introduction

Cesium lead halide perovskite (CsPbX_3_, X = Cl, Br, and I) colloidal quantum dots (QDs), hereafter referred to as PeQD‐X, are considered promising materials for solar cells,^[^ ^1^ ^]^ photodetectors,^[^ ^2^ ^]^ and light‐emitting diodes (LEDs)^[^ ^3^ ^]^ owing to their solution processability, narrow full width at half maximum (FWHM) of luminescence spectra,^[^ ^4^ ^]^ and facile bandgap tunability.^[^ ^5^ ^]^ Moreover, the all‐inorganic PeQD‐X is known to remain stable under ambient and high temperature conditions unlike hybrid methylammonium (MA)‐based MAPbX_3_ materials.^[^ ^6^ ^]^

Particularly, the PeQD‐X can change the bandgap even after the completion of synthesis by exchanging halide anions, thereby rendering the PeQD‐X suitable for LEDs.^[^ ^7^, ^8^ ^]^ Nedelcu et al. reported that the photoluminescence (PL) emission (red, green, and blue colors) of PeQD‐X solutions can be determined based on the ratio of halide anions.^[^ ^9^ ^]^ The emission spectra of PeQD‐X were blue‐shifted with an increased ratio of chloride (Cl^–^) anions, whereas they were red‐shifted when the iodide (I^–^) ratio in the perovskite lattice was increased. However, the color conversion of PeQD‐X occurred only in a solution state without repeating color change because the process of anion exchange required careful choice of precursor solvents and subsequent rinsing processes. During anion exchange, the polar solvents can penetrate PeQD‐X through surface defects generated by the steric hindrance of long‐chain ligands to destabilize the ionic PeQD‐X.^[^ ^10^ ^]^ These solution‐state processes may impair the performance of LEDs based on the PeQD‐X.

Typically, the long‐chain ligands in the exchange process should be replaced with short‐chain ligands to enhance the LED performance.^[^ ^11^, ^12^ ^]^ The PeQD‐X is generally passivated using oleic acid (OA) and oleylamine (OAM) long‐chain ligands. However, long‐chain ligands interrupt the charge transport between each of PeQDs and generate dangling bond defects on the surface, which can deteriorate the LED performance. Therefore, both OAM and OA ligands should be replaced with shorter ligands to ensure accurate exchange and denser passivation of PeQD‐X ligands.

Moreover, patterning the solution based PeQD‐X was particularly difficult. Because the solution spreads on substrate, the micro size bank structures would be needed to confine the emitting layer, and the bank can cause the luminance loss.^[^ ^13^ ^]^ Also, the perovskite materials are very vulnerable for ultraviolet (UV) ray, electron beam (E‐beam), and polar solvent used as developer.^[^ ^14^ ^]^ Hence, direct exposure of UV ray, E‐beam, and developer treatments for perovskite films can cause the degradation of perovskite film quality. Therefore, the patterning process without bank structure and direct exposure of UV ray, E‐beam, and developer treatments are needed for realizing high quality patterned PeQD‐X films.

In this study, we propose a facile all‐in‐one process with simultaneous short ligand and anion exchange process in film‐state for enhancing the LED performance, converting the light‐emitting color, and patterning PeQD‐X LEDs for full‐color displays. The process replaced long‐chain ligands with shorter ones (acetate and butylammonium (BA)), which prevents the penetration of polar solvent at the subsequent steps of rinsing and color‐changing processes. Thus, the improved conductivity of the all‐in‐one process‐treated PeQD‐X films enhances the performance of PeQD‐X LEDs significantly when used as emitting materials.^[^ ^15^ ^]^

Additionally, the proposed all‐in‐one process allows the PeQD‐X to conveniently change the emitting light color of PeQD‐X LEDs by exchanging halide anions, namely BA iodide (BAI) for red, BA bromide (BABr) for green, and a mixture of BA chloride (BACl) and BABr for blue. The green PeQD‐Br LED displayed high brightness of 9084.9 cd m^–2^, low turn‐on voltage (*V*_on_) of 2.63 V, high maximum external quantum efficiency (EQE_max_) of 4.65%, and narrow full width at half maximum (FWHM) of electroluminescence (EL) spectra of 19 nm. Furthermore, other color‐converted LEDs exhibited extremely narrow FWHM of EL spectra of 33 and 19 nm in the case of PeQD‐Br_x_I_3‐x_ (red) and PeQD‐Br_y_Cl_3‐y_ (blue) LEDs, respectively.

Moreover, we produced various paint‐over red/green/blue (R/G/B) patterns using PeQD‐X LEDs by repeating the BA halide (BAX) solution treatments with corresponding halides. Consequently, we fabricated multiple functional PeQD‐X LEDs, namely the R/G/B pixels in a substrate, *one* pixel containing R/G/B colors between a pair of electrodes, and word‐patterned (“KAIST”) LEDs, using the all‐in‐one process. The proposed all‐in‐one process exhibits potential applicability as an effective engineering method for various PeQD‐X‐based optoelectronic devices.

## 2 Results and Discussion

### 2.1 Fabrication Process of All‐in‐One Process for PeQD‐X

**Figure 1:**
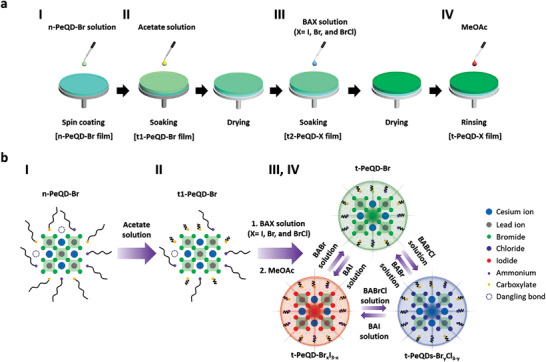
a) Schematic of the fabrication procedure of the all‐in‐one process: I) spin‐coating of n‐PeQD‐Br on a substrate; II) acetate solution treatment for a t1‐PeQD‐Br film; III) butylammonium halide (BAX) solution treatment for a t2‐PeQD‐Br film; IV) rinsing process using MeOAc for a t‐PeQD‐Br film. b) Schematic of the compositional change of PeQD‐X during the all‐in‐one process: I) n‐PeQD‐Br; II) t1‐PeQD‐Br after the acetate solution treatment; III, IV) t‐PeQD‐X after BAX solution treatment and rinsing process.

**Figure** **1** depicts the all‐in‐one fabrication process with simultaneous short ligand and anion exchange for LED devices using PeQD‐Br. As illustrated in Figure 1a‐I, neat‐PeQD‐Br (n‐PeQD‐Br) solutions were spun on a hole transport layer. We arbitrarily selected Br as the halide for our study. Subsequently, the n‐PeQD‐Br films were annealed at 80 °C in a nitrogen‐filled glove box to eliminate the residual solvent. The n‐PeQD‐Br films were then soaked in an acetate solution to replace the long‐chain OA ligands with short‐chain acetate ligands, as depicted in Figure 1a‐II, where t1‐ denotes the PeQD after the first ligand exchange.^[^ ^16^ ^]^ The t1‐PeQD‐Br films were soaked in the BAX (X = Cl, Br, and I) solution for 15 s to exchange the long‐chain OAM ligands with short‐chain BA ligands; t2‐ denotes the PeQD after the second ligand exchange. Simultaneously, the halide anions in t1‐PeQD‐Br were replaced with the target anion X in BAX solution to produce t2‐PeQD‐X, as depicted in Figure 1a‐III. Herein, the anion exchange was performed for color tuning and the halide composition of PeQD‐X can be controlled by treating the different Cl/Br rato or I/Br ratio BAX solutions (Figure S1, Table S1, Supporting Information). After all the ligands and halide anions were replaced, methyl acetate (MeOAc) solutions were used to eliminate impurities that resulted from the all‐in‐one process (Figure 1a‐IV); t‐ denotes the PeQD after the complete exchange and rinsing processes. The experimental conditions are described in detail in the Experimental Section.

Figure 1b depicts the transformation of PeQD‐Br composition in each step during the all‐in‐one process (Figure 1a). As illustrated in Figure 1b‐I, the n‐PeQD‐Br was covered with the OA and OAM long‐chain ligands with dangling bonds that serve as deep surface traps in electronic devices.^[^ ^17^ ^]^ The dangling bonds were generated by the long‐chain OA and OAM ligands because the steric hindrance from the long carbon chains prevented PeQD‐Br from accessing the other OA and OAM ligands. Figure 1b‐II depicts the replacement of OA ligands with acetate ligands.^[^ ^16^ ^]^ During the acetate solution treatment, a few dangling bonds were cured because the acetate ligands with short carbon chains exhibit limited steric hindrance. Figure 1b‐III,IV depict the BAX solution treatment performed to exchange ligands and halide anions of t1‐PeQD‐Br simultaneously. Herein, the OAM ligands are replaced with BA ligands, curing a few of the remaining dangling bonds further. Additionally, the bromide anions in the perovskite lattice can be replaced with the target halide anions in the BAX solution to choose the color of the emitting light. Owing to the all‐in‐one process, most of the OA and OAM ligands were replaced with acetate and BA ligands, which decreased the inter‐dot distance, reduced trap density, and protected the t‐PeQD‐X through dense passivation (Figures S2–S4, Supporting Information). Furthermore, we observed that the emitting light color can be overwritten by repeating the BAX solution treatment, which is discussed in the subsequent sections.

### 2.2 Chemical Composition and Structural Analysis of All‐in‐One Process

**Figure 2:**
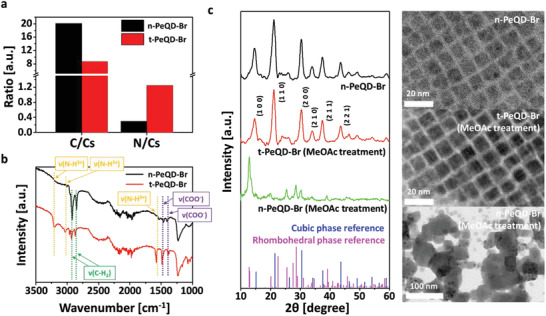
a) Relative ratios of carbon and nitrogen atoms to cesium atoms based on X‐ray photoelectron spectroscopy (XPS) results. The black and red bars represent n‐ and t‐PeQD‐Br, respectively. b) Fourier transform infrared (FT‐IR) spectra of three functional groups are indicated. The black and red lines represent n‐ and t‐PeQD‐Br, respectively. c) X‐ray diffraction (XRD) patterns with black, red, and green lines for n‐ PeQD‐Br, MeOAc treated t‐PeQD‐Br, and MeOAc treated n‐PeQD‐Br, respectively, and transmission electron microscopy (TEM) images of n‐ PeQD‐Br, MeOAc treated t‐PeQD‐Br, and MeOAc treated n‐PeQD‐Br.

We investigated the PeQD‐Br using X‐ray photoelectron spectroscopy (XPS) analysis to identify the extent of exchange between the original long ligands and short‐chain ligands. The ratios of carbon (C) and nitrogen (N) were compared with that of cesium (Cs), which remained constant during the all‐in‐one process. As depicted in **Figure** **2**a, the C/Cs ratio decreased from 20.14 to 8.68. The long‐chain OA and OAM ligands contain eighteen carbons per molecule, whereas the replaced short‐chain acetate and BA ligands comprise two and four carbons per molecule, respectively. Therefore, the decreased C/Cs ratio indicates that PeQD‐Br ligands were replaced with acetate and BA ligands. Conversely, the N/Cs ratio increased from 0.30 to 1.25, indicating that the BA ligands passivated the PeQD‐Br more densely than OAM after the all‐in‐one process. This is because only the OAM and BA ligands comprise nitrogen atoms in the ammonium functional group. Therefore, the decreased C/Cs ratio and increased N/Cs ratio verify that long‐chain ligands of OA and OAM were replaced with short‐chain ligands of acetate and BA. Based on the XPS results, we estimated that the acetate and BA ligands were more densely passivated than OA and OAM by ≈220% and 420%, respectively (Table S2, Supporting Information).

Furthermore, the Fourier transform infrared spectroscopy (FT‐IR) analyses indicate that the long‐chain ligands were replaced. As depicted in Figure 2b, the absorption peaks of the methylene group (C–H_2_) at 2930 and 2860 cm^–1^ decreased after the all‐in‐one process. This indicates that the OA and OAM ligands were replaced with acetate and BA ligands as they comprise lower number of C–H_2_ groups than the OA and OAM ligands. Conversely, the absorption peaks of the ammonium group (N–H_3_^+^) at 3250, 3050, and 1570 cm^–1^ increased after the all‐in‐one process owing to the increased number of NH_3_^+^ groups, implying that BA ligands passivated the PeQD‐Br more densely than OAM. Moreover, the absorption peaks of the carboxylate group (COO^–^) at 1488 and 1400 cm^–1^ increased, verifying the increase in the number of acetate groups passivating the PeQD‐Br.^[^ ^7^, ^16^, ^18^ ^]^

We compared the crystal structure of PeQD‐Br before and after the all‐in‐one process to investigate whether it maintained the cubic phase and shape after the all‐in‐one process. The measurements were obtained using X‐ray diffraction (XRD) and transmission electron microscopy (TEM) analyses. We determined that the average size of n‐PeQD‐Br was 10.56 nm (standard deviation: 1.18 nm) before the all‐in‐one process (Figure S5, Supporting Information). Moreover, the crystal structure and the shape of n‐PeQD‐Br were cubic. However, the cubic structures can be easily damaged by polar solvents owing to the ionic characteristics of PeQD‐X. The repeated exposure of n‐PeQD‐Br to MeOAc solvent partially changed the PeQD‐Br structure from a cubic phase to a rhombohedral phase, which reduced the light emission. Additionally, the Pb‐OA and Pb‐OAM on the PeQD‐Br surface were dismantled, which rendered the disassembled PeQD‐Br prone to fusing with each other. Furthermore, the shapes were transformed (Figure 2c). By contrast, the t‐PeQD‐Br structure retained its cubic phase even after the all‐in‐one process because the densely passivated acetate and BA ligands prevented the polar solvent attack on PeQD‐Br. Consequently, the t‐PeQD‐Br maintained the original cubic shape, validating the robust surface passivation of the all‐in‐one process (Figure 2c).

### 2.3 Device Performance of PeQD‐X Using All‐in‐One Process

**Figure 3:**
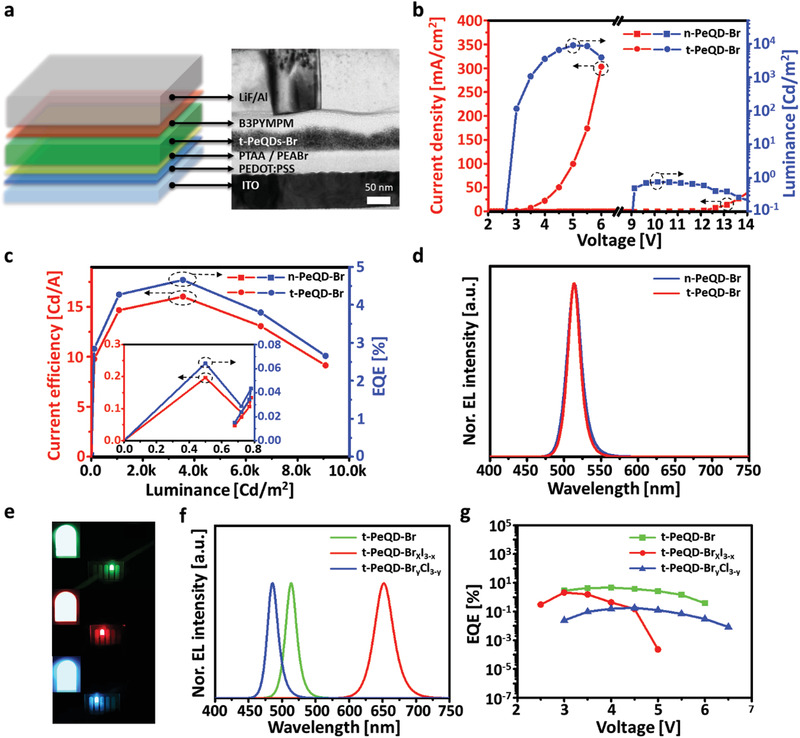
a) Device structure and a cross‐sectional transmission electron microscopy (TEM) image of a t‐PeQD‐Br light‐emitting diode (LED). b) Current density (*J*)–voltage (*V*)–luminance (*L*) characteristics, c) current efficiency (CE)–luminance (*L*)–external quantum efficiency (EQE) characteristics, and d) normalized electroluminescence (EL) spectra of n‐PeQD‐Br and t‐PeQD‐Br LEDs. e) Photographs of t‐PeQD‐X LED devices (top: green (t‐PeQD‐Br), middle: red (t‐PeQD‐Br_x_I_3‐x_), and bottom: blue (t‐PeQD‐Br_y_Cl_3‐y_)). f) Normalized EL spectra, g) EQEs of green (t‐PeQD‐Br), red (t‐PeQD‐Br_x_I_3‐x_), and blue (t‐PeQD‐Br_y_Cl_3‐y_) LEDs.

We compared the device performances of neat and treated PeQD‐Br LEDs to determine the advantage of the proposed all‐in‐one process. The device structure was glass/indium tin oxide (ITO, 70 nm)/poly(3,4‐ethylenedioxythiophene):polystyrene sulfonate (PEDOT:PSS, 40 nm)/poly[bis(4‐phenyl)(2,4,6‐trimethylphenyl)amine] and phenethylammonium bromide (PTAA and PEABr, 10 nm)/neat or treated PeQD‐Br (40 nm)/4,6‐bis(3,5‐di(pyridin‐3‐yl)phenyl)‐2‐methylpyrimidine, 4,6‐bis(3,5‐di‐3‐pyridinylphenyl)‐2‐methylpyrimidine (B3PYMPM, 40 nm)/lithium fluoride (LiF, 1 nm)/aluminum (Al, 150 nm) (**Figure** **3**a). The PTAA was intercalated on the PEDOT:PSS to enhance the hole transport, and the B3PYMPM was used as an electron transport layer (ETL) to efficiently block the injected holes (Figure S6, Supporting Information). Additionally, very thin PEABr layer was used for enhancing adhesion between PeQD‐Br and PTAA layers during the all‐in‐one process (Figure S7, Supporting Information).

As illustrated in Figure 3b, the current density and maximum luminance (*L*_max_) (from 0.8 to 9084.9 Cd m^–2^) were remarkably enhanced, whereas the turn‐on voltage (*V*_on_) reduced significantly from 9.02 to 2.63 V after the all‐in‐one process treatment. As this process perfectly replaced both OAM and OA ligands with shorter ligands, the charge carrier was easily injected into the active layer, which in turn enhanced the amount of injected charge carrier (Figure S8, Supporting Information). Moreover, the short ligands decreased the dangling bonds, which act as surface traps, and increased the radiatively recombination of injected carriers.^[^ ^17^ ^]^ Consequently, the enhanced conductivity and elimination of surface traps improved the current efficiency (CE), external quantum efficiency (EQE), and power efficiency (PE) of t‐PeQD‐Br LEDs, wherein CE_max_ increased from 0.13 to 16.02 Cd A^–1^, EQE_max_ increased from 0.06% to 4.65%, and PE_max_ increased from 0.04 to 13.17 lm W^–1^ (Figure 3c and **Table** **1**). Furthermore, we tried to enhance the EQE of t‐PeQD‐Br LEDs by changing the hole transport layer (HTL) layer. The HTL layers were replaced with PEDOT:PSS and tetrafluoroethylene‐perfluoro‐3,6‐dioxa‐4‐methyl‐7‐octenesulfonic acid copolymer (PFI) mixed layer because the PEDOT:PSS mixed with PFI layer formed a gradually increasing work function, which made possible efficient hole injection.^[^ ^19^ ^]^ Consequently, we could increase the EQE_max_ of t‐PeQD‐Br LEDs to 6.78% (Figure S9, Supporting Information).

**Table 1:** Device performance of n‐ and t‐PeQD‐Br LEDs

	n‐PeQD‐Br	t‐PeQD‐Br
*V*_on_ [V]	9.02	2.63
*L*_max_ [Cd m^–2^]	0.8	9084.9
CE_max_ [Cd A^–1^]	0.13	16.02
EQE_max_ [%]	0.06	4.65
PE_max_ [lm W^–1^]	0.04	13.17

Figure 3d depicts the nearly identical EL spectra along with the unaffected FWHM of neat and treated PeQD‐Br. This implies that the size and bandgap of the PeQD‐Br materials were not affected significantly after the all‐in‐one process. In general,when the PeQDs were fused each other, the EL spectra of PeQD‐Br were red‐shifted because of the increased bandgap and the FWHM of PeQD‐Br EL spectra broadened. Thus, these results demonstrated that the PeQD‐Br LEDs in this study are superior to the previously reported PeQD‐Br LEDs, which were developed using a similar film‐state ligand exchange process.^[^ ^11^ ^]^

Furthermore, we fabricated green, red, and blue LEDs using the all‐in‐one process (Figure 3e). After the acetate solution treatment, the green LEDs were fabricated by using the BABr solution. However, during the BABr solution treatment, the anion exchange did not occur owing to the existence of bromide anions in the n‐PeQD‐Br films; the process only replaced OAM with BA ligands to improve the electrical conductivity of the films. After the BABr solution treatment, the t‐PeQD‐Br LEDs emitted green light with an emission peak of 515 nm, FWHM of 19 nm, and EQE_max_ of 4.65%. Subsequently, the BAI solution treatment exchanged the bromide anions of PeQD‐Br films with iodide anions, transforming the PeQD‐Br films to t‐PeQD‐Br_x_I_3‐x_ films. These t‐PeQD‐Br_x_I_3‐x_ LEDs emitted red light, wherein the emission peak was 650 nm, FWHM was 33 nm, and EQE_max_ was 2.11%. Furthermore, the bromide anions of PeQD‐Br films were exchanged with bromide and chloride anions after the BABrCl solution treatment. The obtained t‐PeQD‐Br_y_Cl_3‐y_ LEDs emitted blue light with an emission peak of 485 nm, FWHM of 19 nm, and EQE_max_ of 0.18% (Figure 3f,g, and Figure S10, Supporting Information).

Additionally, the EL spectra of t‐PeQD‐Br LEDs were highly stable (Figure S11, Supporting information). Also, as shown in Figures S12 and S13 (Supporting Information), the EL spectra of treated blue and red (mixed halides) LEDs were more stable than those of untreated LEDs at the different voltages and continuous operating conditions. Because the all‐in‐one process made the t‐PeQD‐X more perfectly passivated with short chain ligands, the t‐PeQD‐X structure was more stably maintained than the n‐PeQD‐X structure under various operating conditions.^[^ ^20^ ^]^

### 2.4 Facile R/G/B Patterning Process Using All‐in‐One Process

**Figure 4:**
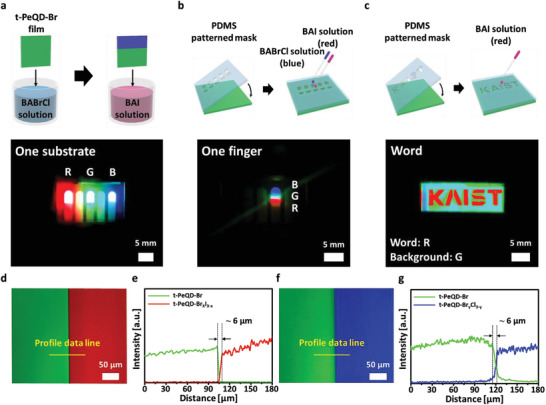
Schematic of patterning processes and photographs of red/green/blue (R/G/B) light‐emitting diodes (LEDs) in one substrate (a). b) A patterned R/G/B LED under a single electrode and c) a patterned word “KAIST” (width = 0.5–1 mm) LED (t‐PeQD‐Br_x_I_3‐x_ (letters, R)/t‐PeQD‐Br (background, G)). d) The confocal laser scanning microscopy (CLSM) images of the boundary in a line‐patterned PeQD film; left: PeQD‐Br, right: PeQDBr_x_I_3‐x_, and e) the line profiles for green and red colors plotted across the yellow line in (d). f) The CLSM images of the boundary in a line‐patterned PeQD film; left: PeQD‐Br, right: PeQDBr_y_Cl_3‐y_, and g) the line profiles for green and blue colors plotted across the yellow line in (f).

We fabricated various R/G/B patterned LEDs to highlight the advantages of the all‐in‐one process. Through the all‐in‐one patternging process, we could realize the various patterned LEDs without direct exposure of ultraviolet light or electron beam to PeQD‐X layer. The proposed process can change the color repeatedly by treating BAX solutions on the film‐state emitting layer. **Figure** **4**a depicts the fabricated red, green, and blue LEDs (placed on three out of five fingers) in a substrate of dimensions 1 × 1 in. by changing the anions on the film‐state layer. The cells were fabricated using a dip‐coating method. After the t‐PeQD‐Br films were prepared, approximately one‐third of one side of the cell was dipped in the BAI solution for red, whereas the other side (approximately one‐third) was dipped in the BABrCl solution for blue, as illustrated in Figure 4a. Herein, the paint‐over patterning was achieved without any masks.

Moreover, we fabricated a single‐finger LED that emitted red, green, and blue colors side‐by‐side and a word‐patterned LED (“KAIST”) of width 0.5–1 mm using *polydimethylsiloxane* (PDMS) patterned masks (Figure 4b,c). Initially, the t‐PeQD‐Br films were prepared to obtain a green background, and the patterned PDMS masks were attached to the films. Subsequently, the BAI and BABrCl solutions were released onto the patterned mask for 15 s to draw patterns with red and blue colors, respectively.

Additionally, we fabricated 300 µm‐width line‐patterned t‐PeQD‐X films using a PDMS mold (Figure S14a, Supporting Information). As depicted in Figure S14b,c (Supporting Information), clearly patterned line widths were observed under confocal laser scanning microscopy (CLSM) analysis. The line boundary was clearly separated with green and red colors. Moreover, the line profile analysis of the CLSM boundary (yellow line in Figure 4d) revealed that the iodide anion was diffused and Figure 4e showed the mixed halides region (≈6 µm). This was further validated by a two‐dimensional PL mapping analysis over the line boundary (Figure S15, Supporting Information). Similarly, Figure 4f indicates that the green and blue boundary was also clearly separated. The line profile of the CLSM boundary (yellow line in Figure 4f) indicates that the undesirable mixed halides region caused by the chloride anion diffusion (≈6 µm) as shown in Figure 4g.

To further verify the potential utilization of the proposed process, we fabricated a green‐emitting LED (PeQD‐Br based) with silver nanowires (AgNWs) as a top electrode (Figure S16, Supporting Information). Subsequently, the device was soaked in the BAI solution, and the LED emitted red light under bias condition. The target halide anions in the BAI solution can permeate through AgNWs. Thus, we demonstrated that the emitting color of PeQD‐X LEDs can be transformed even after the entire device is fabricated.

## 3 Conclusion

To enhance the performance of PeQD‐X LEDs and easily change the emitting light color, we investigated a novel all‐in‐one process in film states. The use of BAX solutions in the proposed all‐in‐one process led to the fabrication of red, green, and blue LEDs based on a single PeQD‐Br solution. This can be attributed to the dense passivation with short‐chain ligands that effectively minimized the degradation of PeQD‐X during the all‐in‐one process. Additionally, the all‐in‐one process in the film‐state aided in fabricating various paint‐over R/G/B patterns in PeQD‐X LEDs owing to the sequential anion exchange process. The experimental results verified the fabrication of multiple functional LED devices, such as R/G/B in a single substrate, R/G/B in a single finger, and a word‐patterned LED. Thus, the proposed process ensures a facile and highly effective ligand exchange that can be potentially applied in various PeQD‐X‐based optoelectronic devices.

## 4 Experimental Section

### Materials

Lead (II) bromide (PbBr_2_, Puratronic, 99.998%, metals basis) and butylammonium hydrochloride (BACl, 98%) were purchased from Thermo Fisher Scientific Chemicals Co., Ltd. The cesium carbonate (Cs_2_CO_3_, trace metals basis, 99.995%), OA (technical grade, 90%), OAM (technical grade, 70%), 1‐octadecene (ODE, technical grade, 90%), acetic acid (≥99.7%), MeOAc (anhydrous, 95%), ethyl acetate (EtOAc, anhydrous, 99.8%), 1‐butanol (BuOH, anhydrous, 99.8%), hexane (laboratory reagent, 95%), octane (anhydrous, 99%), chlorobenzene (CB, anhydrous, 99.8%), *N*,*N*‐dimethylformamide (DMF, anhydrous, 99.8%), BAI (98%), BABr (98%), lead (II) nitrate (Pb(NO_3_)_2_, trace metals basis, 99.999%), lithium fluoride (LiF, trace metals basis, 99.99%), and 4,6‐bis(3,5‐di(pyridin‐3‐yl)phenyl)‐2‐methylpyrimidine, 4,6‐bis(3,5‐di‐3‐pyridinylphenyl)‐2‐methylpyrimidine (B3PYMPM, 99%) were purchased form Sigma‐Aldrich Co., Ltd. Additionally, the PEDOT:PSS aqueous solutions (Clevios AI4083) were purchased from Heraeus, Germany.

### Preparation of Cesium Oleate Precursor

Cesium oleate precursor was prepared by loading Cs_2_CO_3_ (0.5 g), OA (2 mL), and ODE (50 mL) into a 100 mL 3‐neck flask and degassed for 1 h at 120 ℃ until all the Cs_2_CO_3_ reacted with OA. Subsequently, the flask was purged with Ar gas, which was maintained at 100 ℃.

### PeQD‐Br Synthesis

PbBr_2_ (0.69 g) and ODE (50 mL) were loaded into a 100 mL three‐neck flask and degassed at 120 ℃ for 1 h. Additionally, OA (5 mL) and OAM (5 mL) were pre‐heated in N_2_ at 70 ℃. After 1 h of degassing, the flask was purged with Ar, and the pre‐heated OA and OAM were injected. The flask was degassed at 120 ℃ again. When a clear and stable solution was obtained, the flask was purged with Ar, and the temperature was increased to 165 ℃. Subsequently, 8 mL of the previously prepared Cs‐oleate precursor was swiftly injected and stayed for 5 s. Then, the crude solution was cooled down in an ice‐water bath. After synthesis, MeOAc was added to the crude solution in the ratio 1:2 of crude solution:MeOAc in the glove box for purification. This mixture was then separated by centrifugation at 8000 rpm for 10 min. The supernatant was discarded, and the precipitation was dispersed in hexane. Subsequently, the first purified PeQD‐Br solution was centrifuged at 8000 rpm for 10 min, wherein the precipitation was discarded and the supernatant was collected. In the second purification, the MeOAc was added to the supernatant solution with the ratio of supernatant solution:MeOAc = 1:2, and the mixture was separated by centrifugation at 8000 rpm for 10 min. Herein, the supernatant was discarded, and the precipitation was redispersed in octane (6 mL).

### Preparation of Acetate and BAX Solutions

To prepare the acetate solution, Pb(NO_3_)_2_ (20 mg) and acetic acid (0.1 mL) were dissolved in MeOAc (20 mL) while sonicating it for 20 min. The solutions were saturated and centrifuged at 4000 rpm for 5 min to remove excess Pb(NO_3_)_2_. Additionally, BAI (0.1322 g), BABr (0.1013 g), and BACl (0.0721 g) were dissolved in BuOH (20 mL) separately while sonicating it for 20 min to obtain the BAX solutions. Herein, the BAI in BuOH (BAI precursor, 10 mL) was added to EtOAc (20 mL) to obtain the BAI solution, BABr precursor (10 mL) was added to EtOAc (20 mL) to obtain the BABr solution, and BABr (5 mL) and BACl (5 mL) precursors were added to EtOAc (20 mL) to obtain the BABrCl solution.

### Device Fabrication

PEDOT:PSS filtered with 0.45‐µm polyethersulfone (PES) filter was spun onto ITO patterned glasses at 6000 rpm for 60 s, and the films were annealed at 150 ℃ for 30 min. The PTAA solution (5 mg mL^–1^ dissolved in CB) was spin‐coated at 6000 rpm for 60 s, and the films were baked in the glove box at 170 ℃ for 30 min. After cooling down the films, the PEABr solution (10 mg mL^–1^ dissolved in DMF) was spin‐coated at 4000 rpm for 60 s. Subsequently, the purified n‐PeQD‐Br solution was spin‐coated at 2000 rpm for 60 s, and the films were annealed in the glove box at 80 ℃ for 5 min. The acetate solution was then released onto the n‐PeQD‐Br films for 5 s, which were dried while spinning at 2500 rpm for 30 s. Simultaneously, the BAX solutions were treated for 15 s and desiccated while spinning at 2500 rpm for 30 s. The solid‐state ligand‐exchanged PeQD‐X films were rinsed by soaking them in MeOAc for 5 s, and the films dried after spinning for 60 s. Subsequently, the ETL B3PYMPM (40 nm) and top electrode LiF/Al (1 nm/150 nm) were deposited using a thermal evaporator under a high vacuum of 2 × 10^–7^ Torr.

### Characterization

The atomic ratio was obtained using an XPS (K‐alpha, Thermo VG Scientific) analysis, and the morphologies were measured using a TEM (JEM‐ARM200F, JEOL Ltd.). FT‐IR analysis was performed using an FT‐IR spectrometer (iS50 FT‐IR, Nicolet). The crystal structures were measured using XRD (D/MAX 2500, RIGAKU). The morphologies of the PL films were examined using a CLSM (LSM 880, ZEISS). The EL spectra and *J–V–L* characteristics were measured using a spectroradiometer (CS‐2000, Konika Minolta) and programmable source meter (Keithley model 2400).

## Conflict of Interest

The authors declare no conflict of interest.

## Author Contributions

J.K. and K.‐W.S. contributed equally to this work. J.K., K.‐W.S., and J.L. conceived and designed the experiments and prepared the manuscript. J.K. and S.L. synthesized the PeQD‐X and fabricated the LED devices. J.K. and K.‐W.S. performed analyses, such as FT‐IR, XRD, and TEM. J.K. and C.K. performed XPS analysis. J.K. and K.K. fabricated the PDMS patterned masks and obtained measurements of PeQD‐X LED devices. All authors discussed the results and commented on the manuscript.

## Supporting information

Supporting Information

## Data Availability

The data that support the findings of this study are available from the corresponding author upon reasonable request.
