# Supplementary material for: All‐in‐One Process for Color Tuning and Patterning of Perovskite Quantum Dot Light‐Emitting Diodes
Source: Adv Sci (Weinh). 2022 Mar 1;9(13):2200073. doi: 10.1002/advs.202200073 (PMC9069357; doi:10.1002/advs.202200073)
Supplement: Supplementary file 1 — Supporting Information [file ADVS-9-2200073-s001.pdf]

ADVANCED SCIENCE
Open Access

Supporting Information

for *Adv. Sci.*, DOI 10.1002/advs.202200073

All-in-One Process for Color Tuning and Patterning of Perovskite Quantum Dot Light-Emitting Diodes

*Junho Kim, Ki-Won Seo, SeungJae Lee, Kyungmin Kim, Changjo Kim and Jung-Yong Lee**

Supporting Information

All-in-one Process for Color Tuning and Patterning of Perovskite Quantum Dot Light-Emitting Diodes

*Junho Kim, Ki-Won Seo, SeungJae Lee, Kyungmin Kim, Changjo Kim, and Jung-Yong Lee**

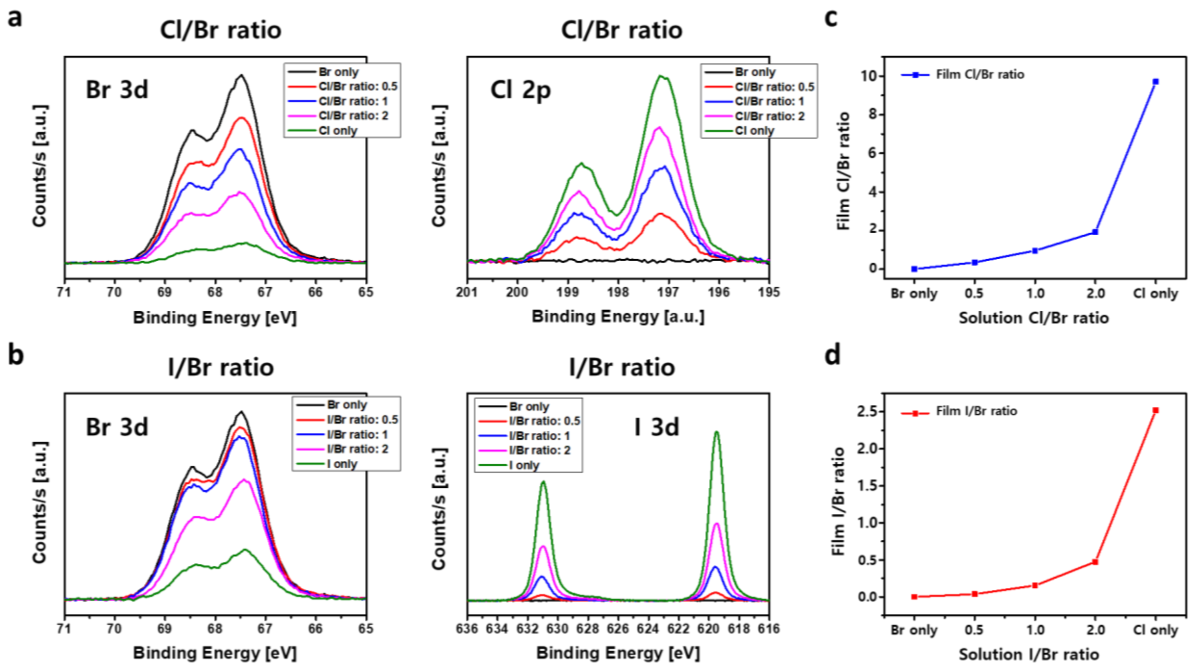

Figure S1. XPS spectra corresponding to Br 3d and Cl 2p following different Cl/Br ratios solution treatments a), and Br 3d and I 3d following different I/Br ratios solution treatments b). c) Cl/Br ratios of the treated films, and d) I/Br ratios of the treated films. The ratio was measured by XPS.

Halide composition analysis of PeQD-X films following the different halide ratio BAX solution treatment:

For the t-PeQD-Br$_y$Cl$_{3-y}$, the Cl/Br ratios of BAX solutions were prepared following Br only, 0.5, 1, 2, and Cl only. Moreover, for the t-PeQD-Br$_x$I$_{3-x}$, the I/Br ratios of BAX solutions were also prepared following Br only, 0.5, 1, 2, and I only.

As shown in Figure S1a, for t-PeQD-Br$_y$Cl$_{3-y}$, the Br 3d peaks (66 ~ 70 eV) were decreased and the Cl 2p peaks (195 ~ 200 eV) were increased as the Cl/Br ratios of BAX solution were

increased. Moreover, as shown in Figure S1b, for t-PeQD-Br_xI_{3-x}, the Br 3d peaks (66 ~ 70 eV) were decreased and the I 3d peaks (616 ~ 634 eV) were increased as the I/Br ratios of BAX solution were increased. In detail, as shown in S1c, S1d, and Table S1, the Cl/Br ratios (from 0.5 to 2) of t-PeQD-Br_yCl_{3-y} were increased almost following the Cl/Br ratios of BAX solution and the Cl only solution can change the Cl/Br ratio until 9.7. On the other hand, the I/Br ratios (from 0.5 to 2) of t-PeQD-Br_xI_{3-x} were increased following the tendency that the more I^- anions were replaced as I/Br ratio of BAX solution increase. The I only solution can replace the I/Br ratio until 2.5. It can be seen that the difference comes from the halide anion size because the larger I^- anions were less efficient to anion exchange than the smaller Cl^- anions.[1]

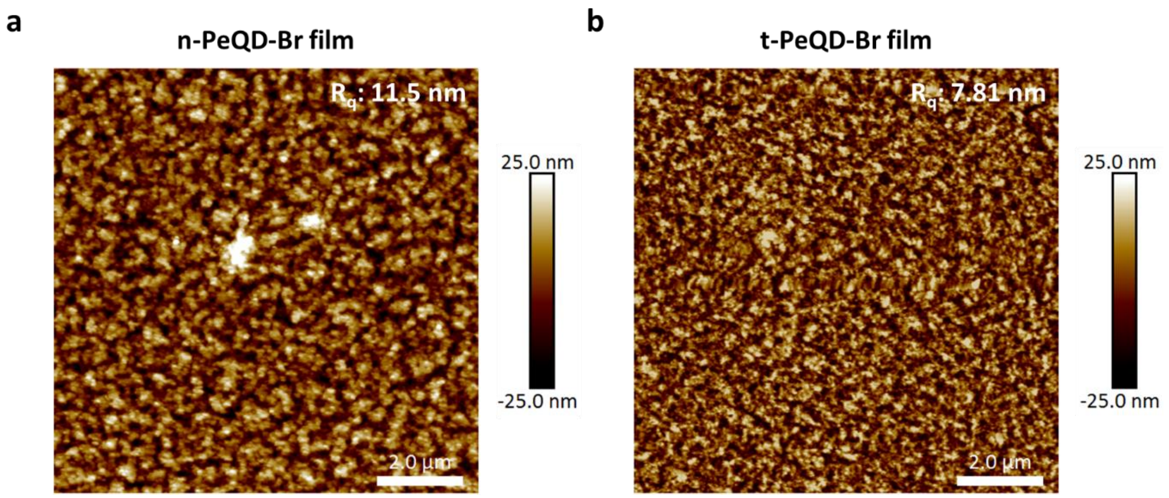

Figure S2. AFM images of n-PeQD-Br film (surface roughness (R_q): 11.5 nm) a) and t-PeQD-Br film (R_q: 7.81 nm) b). The scale bar: 2.0 μm.

Morphology of n- and t-PeQD-Br films:

As shown in Figure S2a and S2b, the morphologies of n- and t-PeQD-Br films were different. The surface roughness (R_q) of n-PeQD-Br film and t-PeQD-Br film were 11.5 nm and 7.81 nm, respectively. As shown in Figure 2a and 2b, the ligand exchange converted from long carbon chain ligands to short carbon chain ligands were investigated using XPS and Fourier transform infrared (FT-IR) analyses. Consequently, the all-in-one process replacing with short chain ligands can decrease the inter-dot spacing, which made the t-PeQD-Br films more uniform and compact.[2]

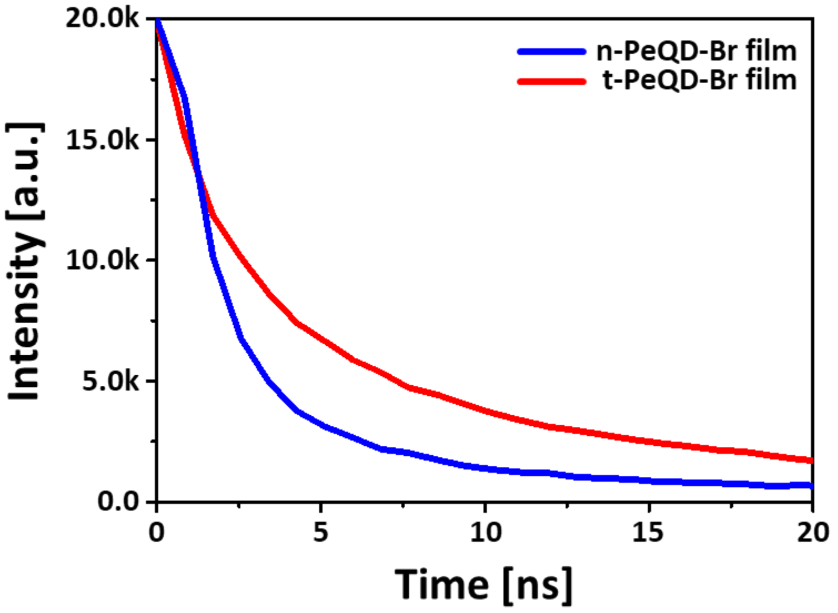

Figure S3. Time-resolved photoluminescence (TRPL) decay curves of n-PeQD-Br (blue line) and t-PeQD-Br (red line).

Time-resolved photoluminescence (TRPL) analysis to investigate trap density reduction after all-in-one process:

The Figure S3 shows TRPL decay curve of n- and t-PeQD-Br films. The TRPL decay curves were fitted with bi-exponential decay model to obtain the average PL decay time ($\tau_{avg,PL}$). The $\tau_{avg,PL}$ was estimated using Equation S1.[3]

$$\tau_{avg,PL} = \frac{A_1 * {\tau_1}^2 + A_2 * {\tau_2}^2}{A_1 * \tau_1 + A_2 * \tau_2} \quad \text{(S1)}$$

It was found that the $\tau_{avg,PL}$ of n- and t-PeQD-Br was 2.26 ns and 5.60 ns, respectively. The $\tau_{avg,PL}$ was increased after all-in-one process, which proved that the surface defects of t-PeQD-Br were decreased by dense passivation (Figure 2a and 2b).[4, 5] Consequently, the trap density was decreased after all-in-one process because of dense passivation of t-PeQD-Br.

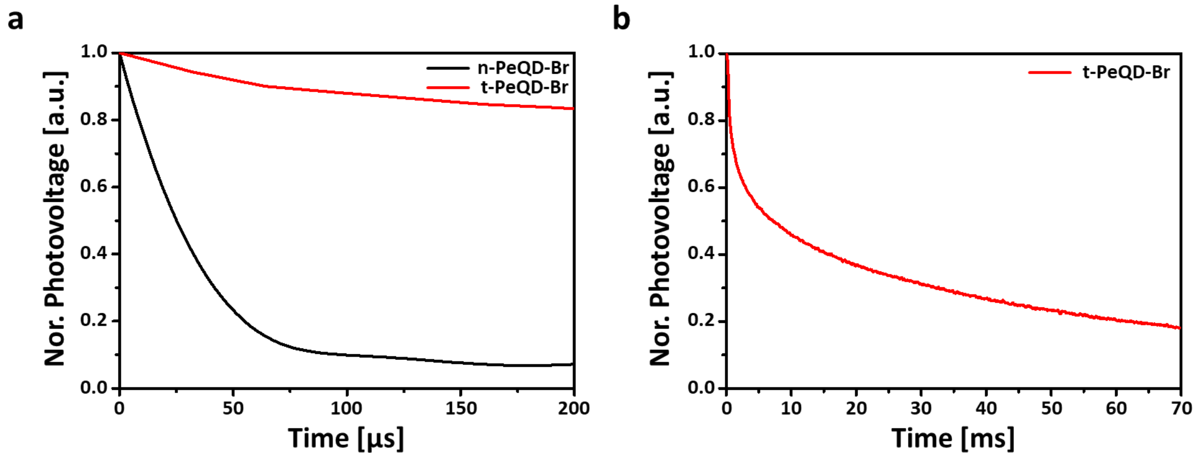

Figure S4. a) Transient photovoltage (TPV) measurements of n-PeQD-Br (black line) and t-PeQD-Br (red line) at μs scale. b) TPV measurement of the t-PeQD-Br at ms scale. The devices were modified to photovoltaic structure to precisely detect the photovoltage.

Transient photovoltage (TPV) measurement to demonstrate trap density reduction after all-in-one process:

The TPV measurement shows transient photovoltage for observing the recombination properties of n- and t-PeQD-Br layers (Figure S4a and S4b). The devices were fabricated using a photovoltaic structure for easily detecting photovoltage, which was poly(3,4-ethylenedioxythiophene):polystyrene sulfonate (PEDOT:PSS, 40 nm)/poly[bis(4-phenyl)(2,4,6-trimethylphenyl)amine] and phenethylammonium bromide (PTAA and PEABr, 10 nm)/n- or t-PeQD-Br (40 nm)/ fullerene, (C_{60}, 20 nm)/bathocuproine (BCP, 5 nm)/copper (Cu, 120 nm). The TPV measurements were fitted with bi-exponential decay model. The average lifetime ($\tau_{avg,TPV}$) was estimated using the Equation S2.[6]

$$\tau_{avg,TPV} = \frac{A_1 * \tau_1 + A_2 * \tau_2}{A_1 + A_2} \quad \text{(S2)}$$

$\tau_{avg,TPV}$ of n- and t-PeQD-Br was 30.02 μs and 12.65 ms, respectively. Therefore, the t-PeQD-Br had longer average recombination lifetime than n-PeQD-Br. It can be inferred that the well-passivated PeQD-Br surface by the all-in-one process decreased trap density.

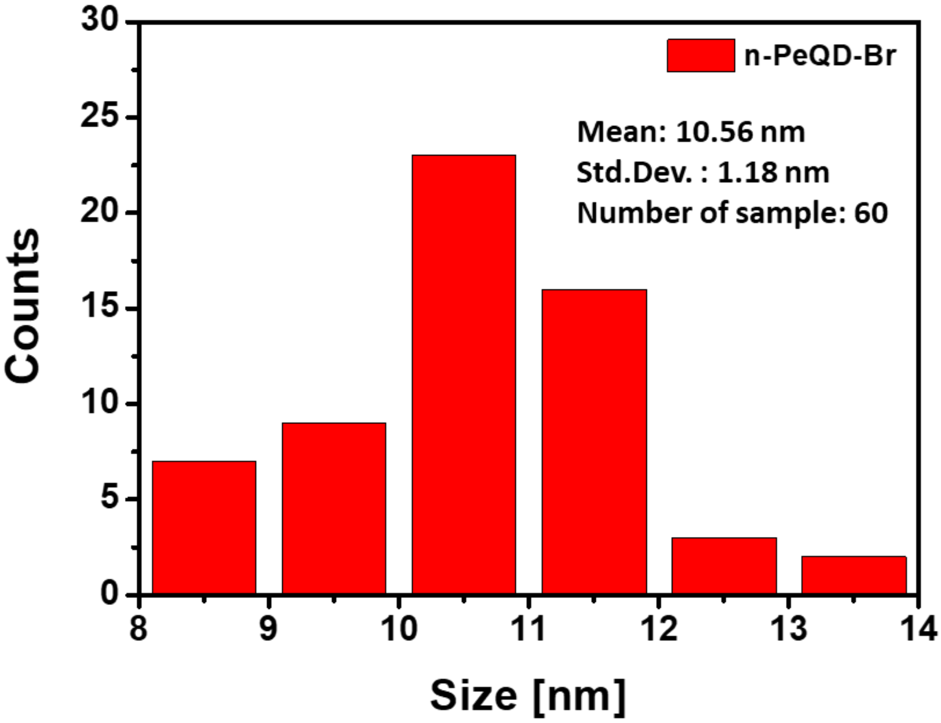

Figure S5. Size distribution histogram of synthesized perovskite colloidal quantum dots (QDs) (cesium lead bromide perovskite QDs: PeQD-Br). The total number of PeQD-Br was 60.

Size distribution of n-PeQD-Br:

The size distribution of the as-fabricated PeQD-Br was obtained from the transmission electron microscopy (TEM) images. The average size of the PeQDs was 10.56 nm and the standard deviation was 1.18 nm.

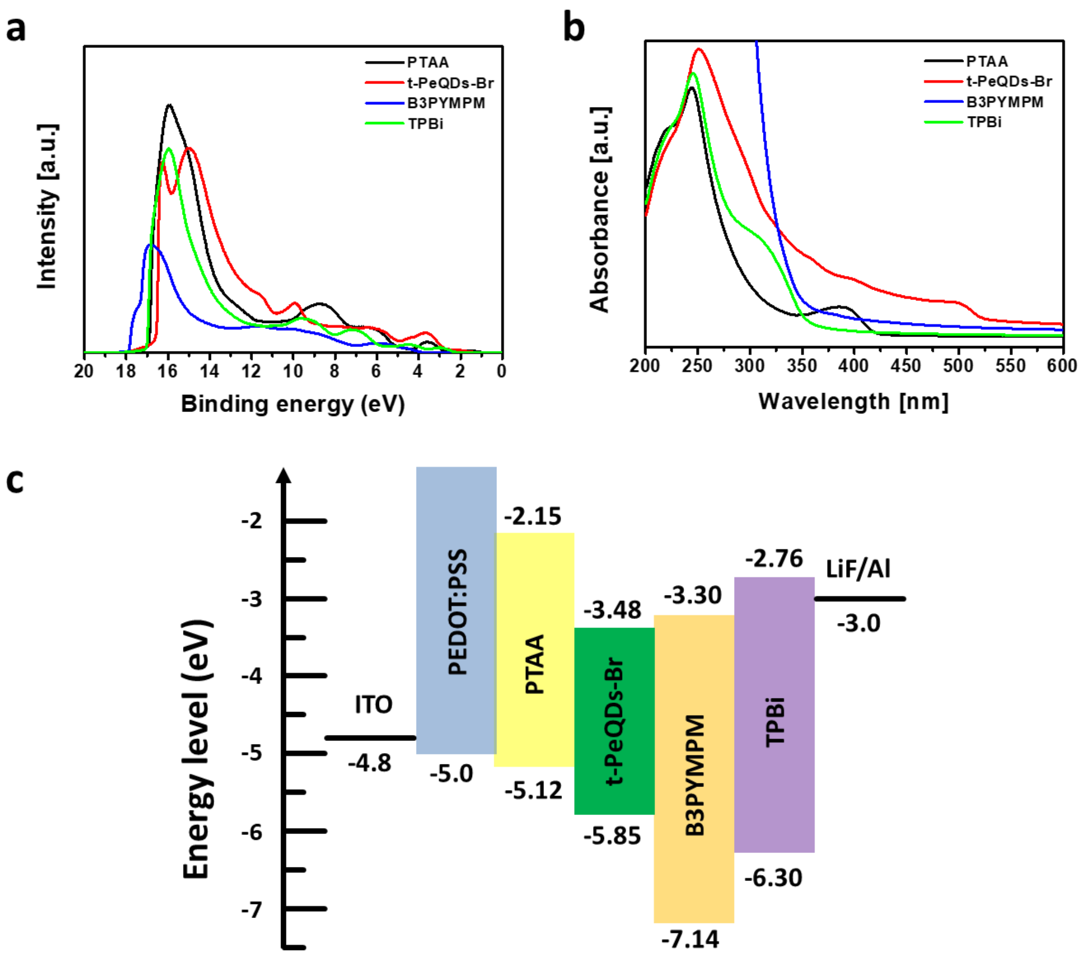

Figure S6. a) Ultraviolet photoelectron spectroscopy (UPS) spectra, b) ultraviolet-visible spectrophotometer (UV-vis) absorption spectra of PTAA, t-PeQDs-Br, B3PYMPM, and TPBi thin films, and c) energy level diagram of t-PeQD-Br LEDs.

Device structure of PeQD-Br LED for enhanced performance:

The energy level of each layer was estimated using ultraviolet photoelectron spectroscopy (UPS) (Figure S6a) and absorption spectra (Figure S6b). The energy levels of the ITO, PEDOT:PSS, and LiF/Al layers were obtained from previous reports[7] (Figure S6c). The PTAA layer had a lower highest occupied molecular orbital (HOMO) level (5.12 eV) than the PEDOT:PSS layer (5.0 eV). Holes could easily be injected from the PEDOT:PSS to the t-PeQDs-Br layer through intercalating the PTAA layer between the PEDOT:PSS and t-PeQDs-Br layers.

Furthermore, the B3PYMPM had lower HOMO than those of the TPBi layer and the lowest unoccupied molecular orbital (LUMO) levels. The lower HOMO level of the B3PYMPM layer resulted in the injected hole remaining in the t-PeQDs-Br layer by blocking the holes.

Moreover, the lower LUMO level of the B3PYMPM layer was beneficial for electron injection from LiF/Al to the t-PeQDs-Br layer.

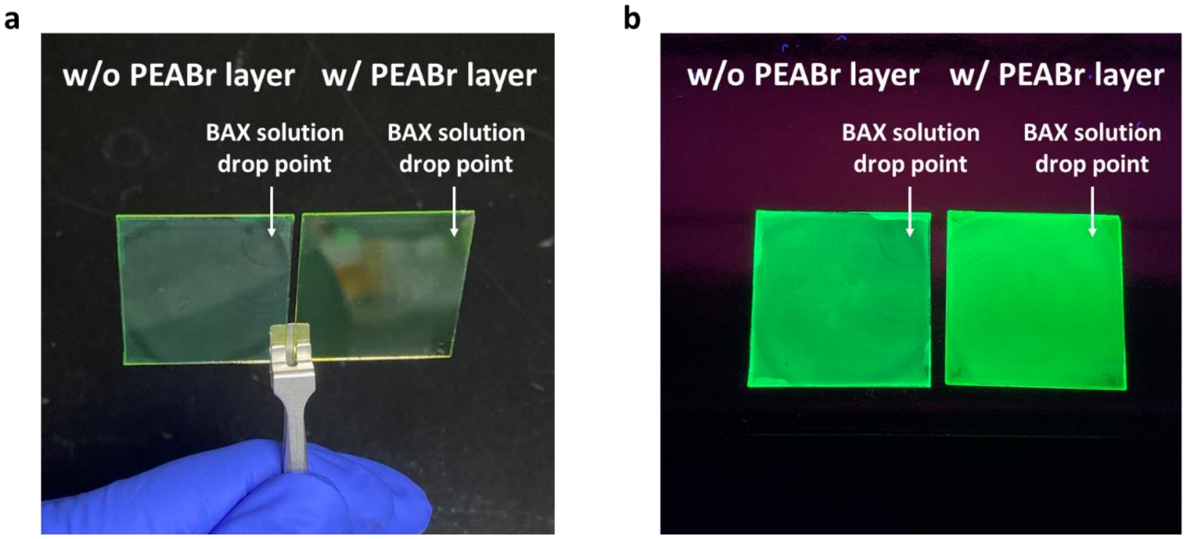

Figure S7. Images of all-in-one processed PeQD-Br film with (w/) and without (w/o) thin PEABr layer. Under fluorescent lamp a), and under ultra-violet (UV) lamp b).

Enhancement of PeQD-Br layer adhesion with PTAA layer by intercalating very thin PEABr layer:

Very thin PEABr layer was used for modifying interface between PeQD-Br and PTAA layers. The PEABr layer increased adhesion between PeQD-Br and PTAA layers during the all-in-one process. As shown in Figure S7a, the PeQD-Br film without thin PEABr layer was damaged following the BAX solution spreading direction. Conversely, the PeQD-Br film using the thin PEABr layer was intact because the very thin PEABr layer enhanced adhesion between the PeQD-Br and PTAA layers (Figure S7b). In detail, the phenyl part of PEABr enhanced the adhesion with PTAA layer because the PTAA was hydrophobic. On the other hand, the ammonium part of PEABr increase the adhesion with PeQD-Br layer by passivating the PeQD-Br surface.[8]

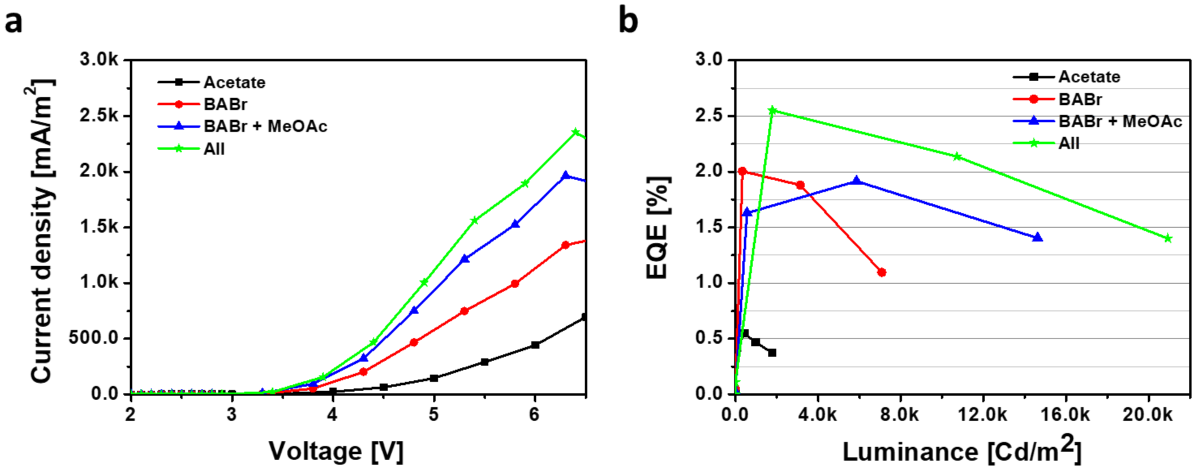

Figure S8. a) Current density (*J*)–voltage (*V*) curves and b) EQE–luminance (*L*) curves of acetate, BABr, BABr+MeOAc (rinsing), and all-in-one process (all) treated PeQD-Br LEDs.

Role of each step of all-in-one process:

The PeQD-Br LEDs were fabricated by treating the film with acetate solution only, BABr solution only, BABr solution and MeOAc rinsing only, and all steps to demonstrate the validity of each step in the all-in-one process. As illustrated in Figure S8a, the current density was increased in the treatment order of $Pb(NO_3)_2$ < BABr < BABr+MeOAc < all steps. Similarly, the external quantum efficiency (EQE) was increased in the treatment order of $Pb(NO_3)_2$ < BABr ≈ BABr + MeOAc < all steps (Figure S8b). When comparing the *J*–*V* and EQE–*L* curves of the acetate solution treated with the BABr solution-treated PeQD-Br LEDs, the BABr solution treatment appeared to have a greater impact on enhancing the PeQD-Br LED performance than the acetate solution treatment. However, the acetate solution treatment was required in addition to the BABr solution and MeOAc rinsing processes to achieve the highest PeQD-Br LED performance (Figure S8b).

As the acetate solution treatment replaced the long-chain oleic acid (OA) with acetate ligands, the OA ligands eliminated were more effectively and the short-chain acetate ligands passivated the PeQDs-Br more densely. Furthermore, because the MeOAc rinsing process eliminated the insulating impurities that were byproducts of the preceding ligand exchange processes, the charge carriers were easily injected into the PeQDs-Br layer, thereby resulting in higher luminance (Figure S8b).

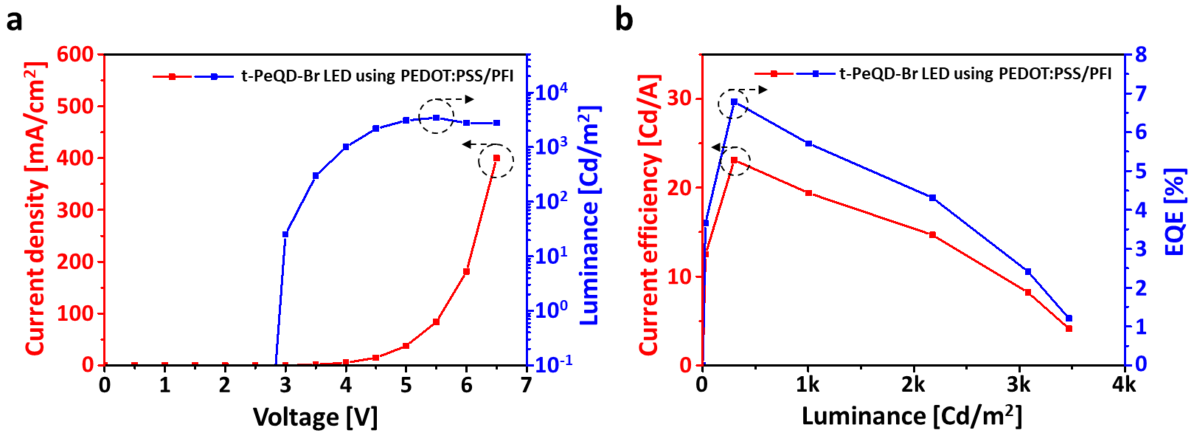

Figure S9. a) Current density (*J*)-voltage (*V*)-luminance (*L*) characteristics, and b) current efficiency (CE)-luminance (*L*)- external quantum efficiency (EQE) characteristics of t-PeQD-Br LEDs using PEDOT:PSS mixed with PFI layer as a hole transport layer.

Maximum external quantum efficiency (EQE_{max}) enhancement of t-PeQD-Br LEDs by optimizing hole transport layer (HTL):

We further optimized the t-PeQD-Br LED device structure by changing hole transport layer. The optimized structure was PEDOT:PSS mixed with tetrafluoroethylene-perfluoro-3,6-dioxa-4-methyl-7-octenesulfonic acid copolymer (PFI)/t-PeQD-Br/B3PYMPM/LiF/Al. As a result, the maximum luminance (L_{max}) was 3469.3 Cd m^{-2} and the maximum current efficiency (CE_{max}) was 19.40 Cd A^{-1}. Furthermore, we were able to renew the EQE_{max} record through t-PeQD-Br LED by using film-state ligand exchange process to 6.78% (Figure S9a and S9b).

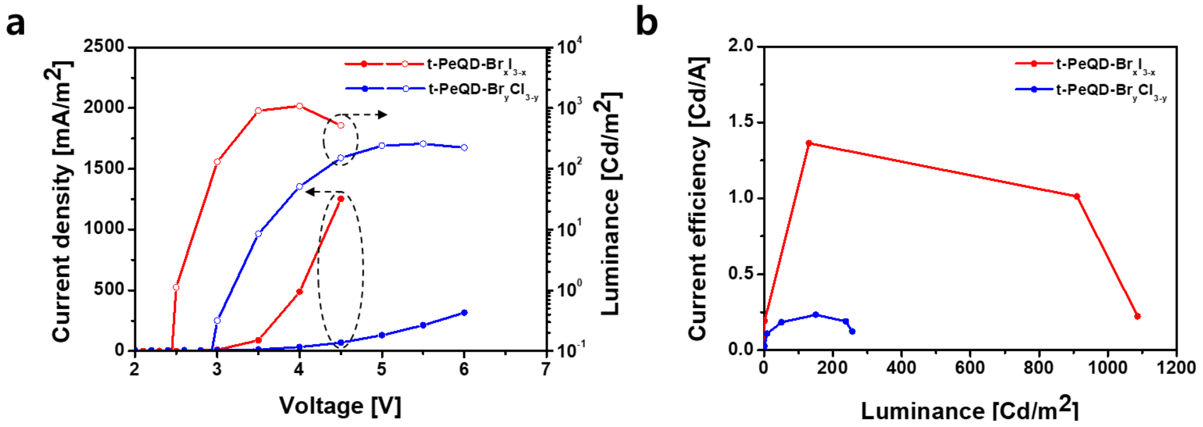

Figure S10. a) *J*–*V*–*L* characteristics and b) CE–*L* characteristics of t-PeQD-Br_xI_{3-x} (red) and t-PeQD-Br_yCl_{3-y} (blue) LEDs.

Performance of t-PeQD-Br_xI_{3-x} (red) and t-PeQD-Br_yCl_{3-y} (blue):

The maximum luminance (L_{max}) and maximum current efficiency (CE_{max}) of the PeQD-Br_xI_{3-x} LEDs were 1086.3 Cd/m^2 and 1.36 Cd/A, respectively. Furthermore, the L_{max} and CE_{max} values for the PeQD-Br_yCl_{3-y} LED devices were 256.2 Cd/m^2 and 0.23 Cd/A, respectively.

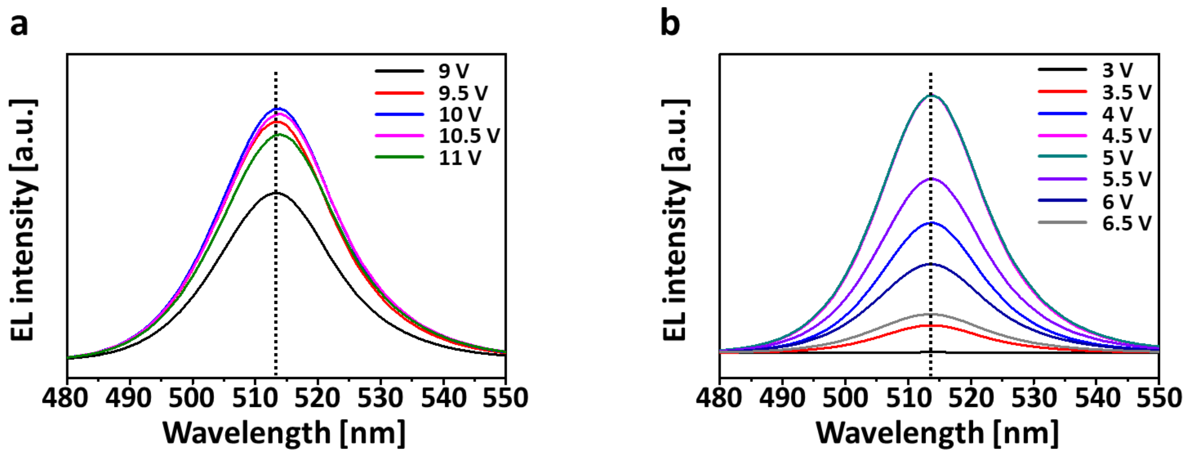

Figure S11. Electroluminescence (EL) spectra of n-PeQD-Br LED a) and t-PeQD-Br LED b).

The stability of n- and t-PeQD-Br LEDs at different voltages:

We compared the electroluminescence (EL) spectra of n- and t-PeQD-Br light emitting diodes (LEDs) to observe spectral stability at the different driving voltages. Because the n-PeQD-Br LEDs were turned on at 9 V, the EL spectra of n-PeQD-Br LEDs were measured between 9 to 11 V. On the other hand, the t-PeQD-Br LEDs were turned on at 3 V, and therefore, the EL spectra of t-PeQD-Br LEDs were observed from 3 to 6.5 V.

As shown in Figure S11a, the EL spectra of n-PeQD-Br LED were slightly red shifted as increasing over 10.5 V. Because the n-PeQD-Br films have long carbon chain ligands, the n-PeQD-Br films could be degraded by burn caused by high resistance of the films at high voltages.[9] On the other hand, the t-PeQD-Br films have low resistance owing to short carbon chain ligands. Hence, the carriers were more easily injected and transported than n-PeQD-Br films, which decreased driving voltage of the t-PeQD-Br LED. Therefore, the EL spectra of t-PeQD-Br LED were not changed in the entire driving voltages (Figure S11b). Consequently, the EL spectra of t-PeQD-Br LED at various driving voltages were more stable than the n-PeQD-Br LED.

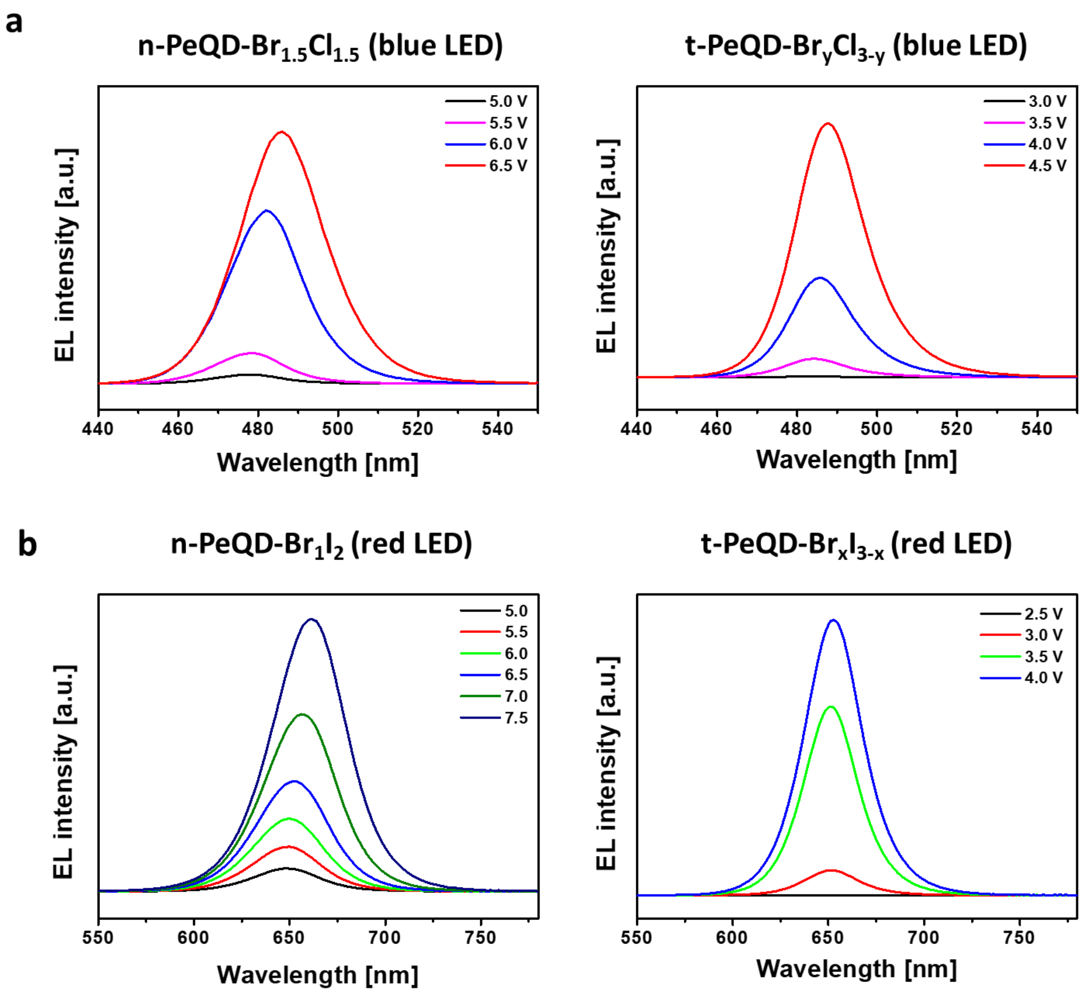

Figure S12. The electroluminescence (EL) spectra of n-PeQD-$Br_{1.5}Cl_{1.5}$ LEDs operating from 5.0 to 6.5 V and t-PeQD-Br_yCl_{3-y} LEDs operating from 3.0 to 4.5 V a), n-PeQD-Br_1I_2 LEDs operating from 5.0 to 7.5 V and t-PeQD-Br_xI_{3-x} LEDs operating from 2.5 to 4.0 V b).

The stability of n- and t-PeQD-X LEDs at different voltages:

To explore the electroluminescence (EL) spectral change of blue and red PeQD-X LEDs at different voltages, we compared the EL spectra of n-PeQD-$Br_{1.5}Cl_{1.5}$ with those of the t-PeQD-Br_yCl_{3-y} LEDs for blue LEDs and the EL spectra of the n-PeQD-Br_1I_2 with those of the t-PeQD-Br_xI_{3-x} LEDs for red LEDs. The n-PeQD-$Br_{1.5}Cl_{1.5}$ and n-PeQD-Br_1I_2 LEDs were turned on at 5 V and the LED performance was very low. In contrast, the t-PeQD-Br_yCl_{3-y} and t-PeQD-Br_xI_{3-x} LEDs passivated with short chain ligands were well operated at low voltages and the turn on voltage of LEDs were 3.0 V and 2.5 V, respectively.

As shown in Figure S12a, the EL spectra of n-PeQD-$Br_{1.5}Cl_{1.5}$ LEDs were red shifted from 478.0 (at 5.0 V) to 486.0 nm (at 6.5 V) and the difference was 8 nm. In contrast, the EL spectra of t-PeQD-Br_yCl_{3-y} LEDs were shifted from 484 (at 3.0 V) to 487.5 nm (at 4.5 V) and

the peak shift was only 3.5 nm. Moreover, the EL spectra of n-PeQD-Br_1I_2 LEDs for red light were also red shifted from 648.5 nm (at 5.0 V) to 662.0 nm (at 7.5 V) and the peaks were moved by 13.5 nm. On the other hand, the EL spectra of t-PeQD-Br_xI_{3-x} LEDs were shifted from 652.5 nm (at 2.5 V) to 652.5 (at 4.0 V) and the peaks were almost unchanged (Figure S12b). It was well known that the dense passivation with ligands can make the perovskite crystal structure more stable.[3, 4] Therefore, the structures of t-PeQD-Br_yCl_{3-y} and t-PeQD-Br_xI_{3-x} can be stably maintained at different voltages because the t-PeQD-Br_yCl_{3-y} and t-PeQD-Br_xI_{3-x} were passivated densely with short chain ligands by all-in-one process.

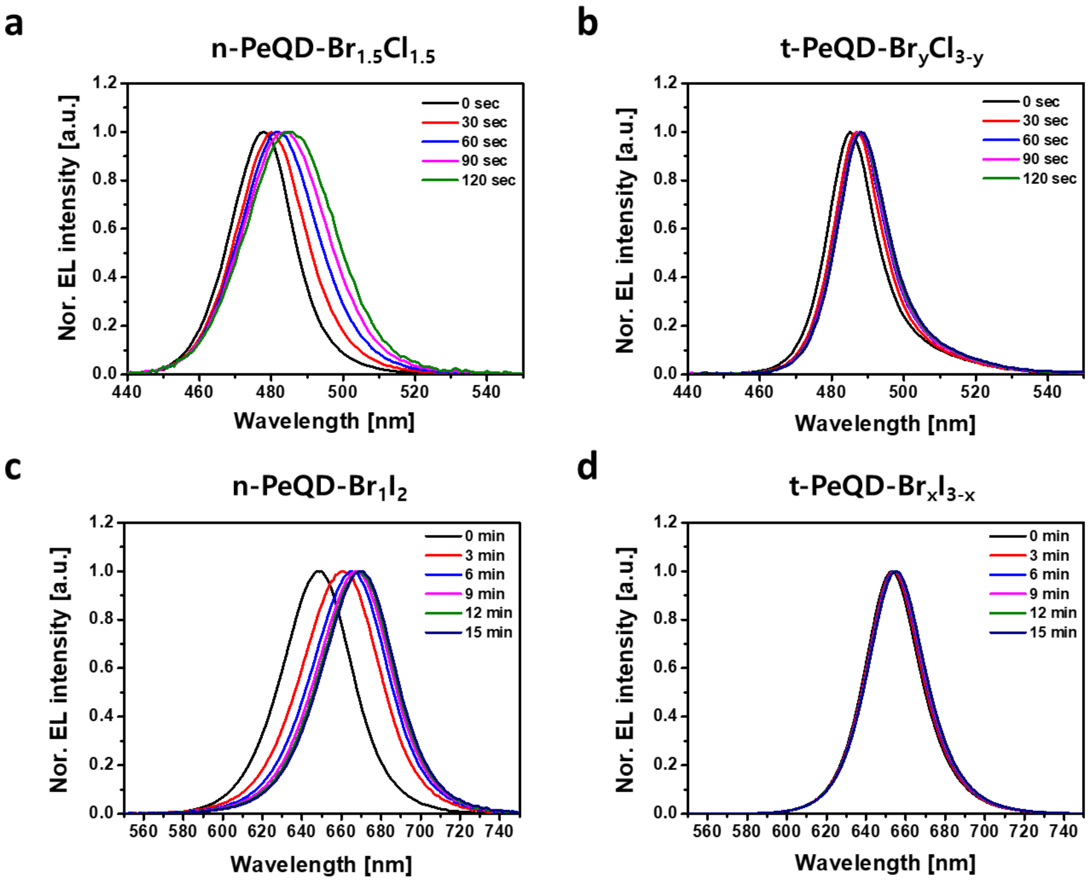

Figure S13. The electroluminescence (EL) sepctra of n-PeQD-$Br_{1.5}Cl_{1.5}$ LEDs operating at 5.5 V for 120 sec a), t-PeQD-Br_yCl_{3-y} LEDs operating at 3.5 V for 120 sec b), n-PeQD-Br_1I_2 LEDs operating at 5.5 V for 15 min c), t-PeQD-Br_xI_{3-x} LEDs operating at 3.0 V for 15 min d).

The operating stability of n- and t-PeQD-X LEDs:

We compared the EL spectra of n-PeQD-$Br_{1.5}Cl_{1.5}$ LEDs with those of the t-PeQD-Br_yCl_{3-y} LEDs converted from n-PeQD-Br films, and the EL spectra of n-PeQD-Br_1I_2 LEDs with those of t-PeQD-Br_xI_{3-x} LEDs converted from n-PeQD-Br films to confirm the stability of emission spectra during the continuous operation condition.

First of all, the n-PeQD-$Br_{1.5}Cl_{1.5}$ LEDs and n-PeQD-Br_1I_2 LEDs did not operate at the same t-PeQD-X film thickness because the long chain OA and OAM ligand interrupted carrier injection. Hence, the n-PeQD-$Br_{1.5}Cl_{1.5}$ and n-PeQD-Br_1I_2 LEDs were fabricated by diluting the solution at a half concentration. Nevertheless, the n-PeQD-$Br_{1.5}Cl_{1.5}$ and n-PeQD-Br_1I_2 LEDs were turned on at 5 V and the LED performance was very low. On the other hand, the t-PeQD-Br_yCl_{3-y} and t-PeQD-Br_xI_{3-x} LEDs were well operating at low voltages and the LEDs were turned on at 3.0 V and 2.5 V, respectively. Hence, the operation stability of n-PeQD-

$Br_{1.5}Cl_{1.5}$ and n-PeQD-Br_1I_2 LEDs was measured at 5.5 V and the t-PeQD-Br_yCl_{3-y} and t-PeQD-Br_xI_{3-x} LEDs were measured at 3.5 V and 3.0 V, respectively.

The Figure S13a and S13b show that the electroluminescence (EL) spectral change of n-PeQD-$Br_{1.5}Cl_{1.5}$ and t-PeQD-Br_yCl_{3-y} LEDs for 2 minutes. The EL spectra of n-PeQD-$Br_{1.5}Cl_{1.5}$ LEDs were red-shifted from 477.5 nm to 486.0 nm (difference: 8.5 nm) for 2 minutes. On the other hand, the EL spectra of t-PeQD-Br_yCl_{3-y} LEDs were shifted only from 485.0 nm to 488.5 nm (difference: 3.5 nm) for 2 minutes. Moreover, the EL spectra of n-PeQD-Br_1I_2 and t-PeQD-Br_xI_{3-x} LEDs were measured for 15 minutes because the red LEDs were generally more stable under operation condition. The peaks of n-PeQD-Br_1I_2 LED spectra were red-shifted from 648.0 nm to 670.5 nm (difference: 22.5 nm) for 15 minutes. (Figure S13c) On the other hand, the peaks of t-PeQD-Br_xI_{3-x} LED spectra were moved from 653.0 nm to 655.5 nm (difference: 2.5 nm) for 15 minutes (Figure S13d).

The modified (or treated) LEDs were more stable than n-PeQD-$Br_{1.5}Cl_{1.5}$ and n-PeQD-Br_1I_2 LEDs because the dense ligand passivation of all-in-one process maintained the perovskite structure during the operation.[3, 4]

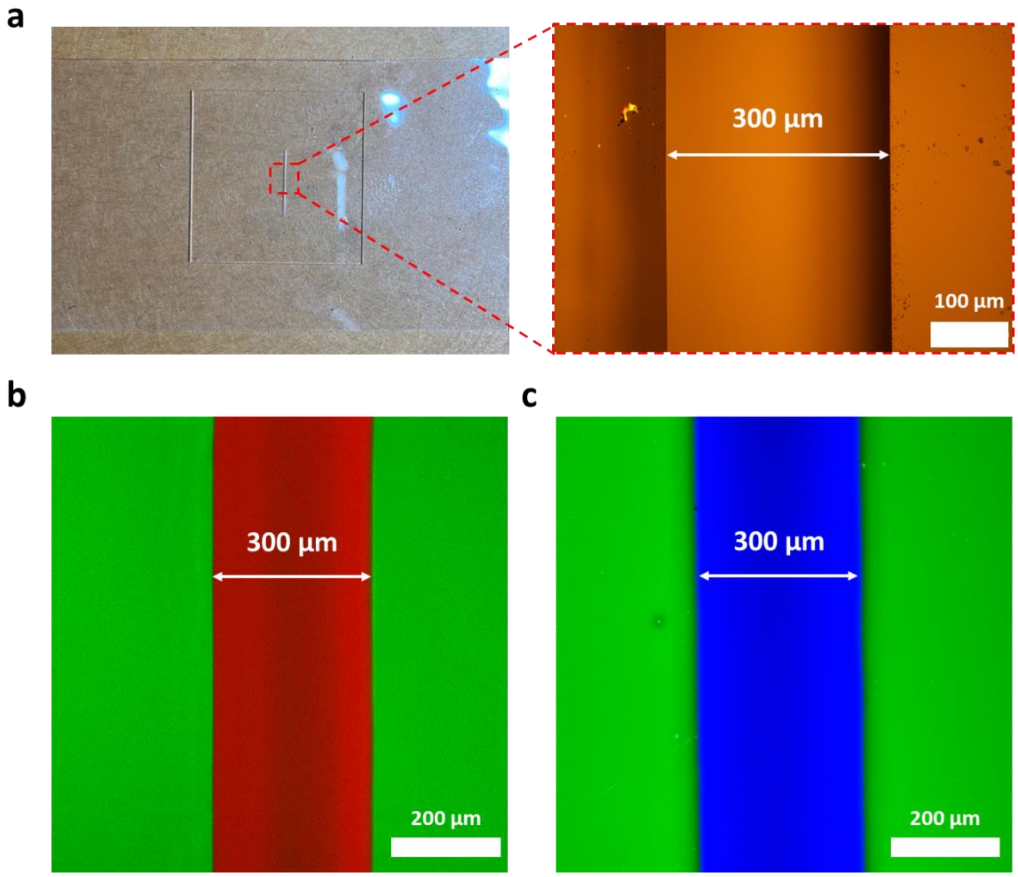

Figure S14. a) Photograph and optical microscope images of line-patterned PDMS mask (width: 300 µm), and CLSM images of line-patterned PeQD film using patterned PDMS mask: b) center line: PeQD-Br_xI_{3-x}; background: PeQD-Br and c) center line: PeQD-Br_yCl_{3-y}; background: PeQD-Br.

Clearly patterned PeQD-X films:

As illustrated in Figure S14a, a line-patterned PDMS mask (line width: 300 µm) was prepared. Using the PDMS mask on a PeQD-Br (green) film, BAI and BABrCl solutions were dropped onto the t-PeQD-Br films for red and blue lines, respectively.

The width of the mask was compared with the patterned red and blue lines using confocal laser scanning microscopy (CLSM) (width: 300 µm). Consequently, the PDMS mask width was the same as the red and blue line widths of the patterned PeQD films (Figure S14b and S14c).

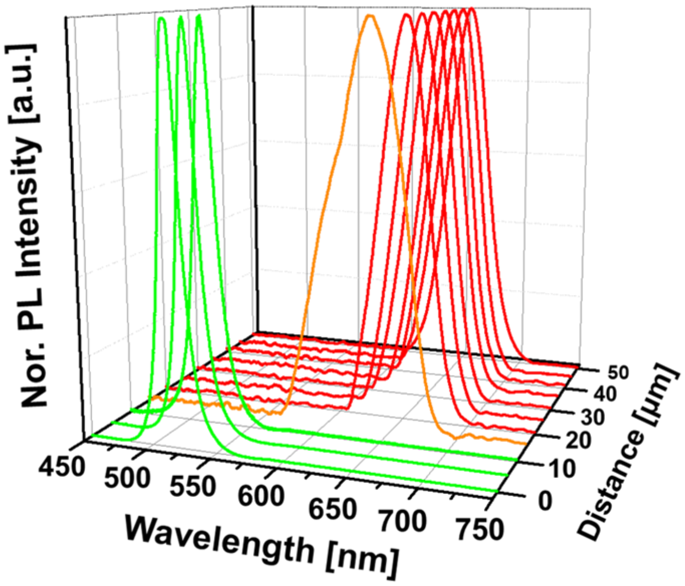

Figure S15. PL mapping data across the boundary between green (t-PeQD-Br) and red (t-PeQD-Br_xI_{3-x}).

Halide anion diffusion over boundary:

Figure S15 depicts the photoluminescence (PL) mapping across the boundary between the PeQD-Br and PeQD-Br_xI_{3-x} regions. The PL in the PeQD-Br_xI_{3-x} region was corrected, because when the PeQD-Br_xI_{3-x} was exposed to a strong laser, the well-oriented mixed halides could be divided into iodide-rich and bromide-rich domains.[10] It can clearly be observed from Figure S15 that the iodide anion was diffused less than 10 μm when the PeQD-Br_xI_{3-x} solution was dropped onto the PeQD-Br layer.

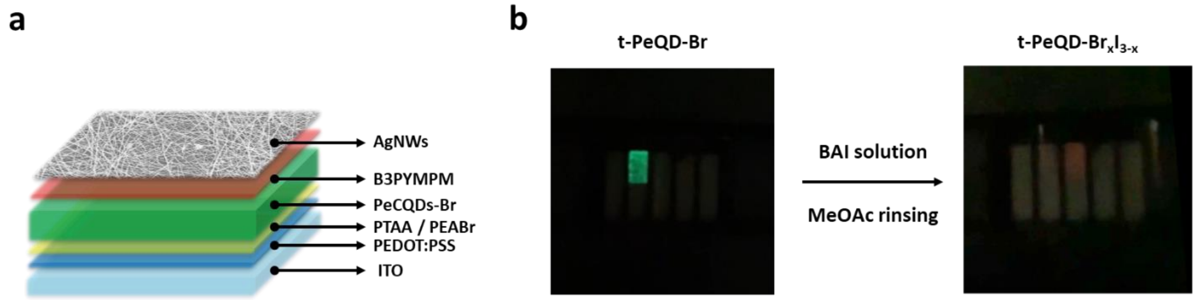

Figure S16. a) PeQD LED device structure using AgNWs as top electrode, and b) photographs of green (t-PeQD-Br) and red (color converted, t-PeQD-Br$_x$I$_{3-x}$) LEDs.

PeQD LED color conversion following completion of full device:

A green-emitting LED with a structure of glass / ITO / PEDOT:PSS / PTAA / PEABr / t-PeQDs-Br / B3PYMPM / LiF / AgNWs was fabricated. Following fabrication of the t-PeQD-Br LED, the LED was soaked in a BAI solution, which could penetrate the t-PeQD-Br layer passing through the AgNW top electrode. As a result, the t-PeQD-Br layer was transformed into a t-PeQD-Br$_x$I$_{3-x}$ layer, which emitted red light under electrical bias.

Table S1. The atomic ratios of n-, and t-PeQD-Br corresponding to Figure S1. The atomic ratios were measured by XPS.

	Cs [%]	Cl [%]	Br [%]	I [%]
Br only	23.09	0.13	76.17	0.07
Cl/Br ratio: 0.5	24.93	19.50	55.36	0.21
Cl/Br ratio: 1	25.22	36.52	38.07	0.19
Cl/Br ratio: 2	26.96	47.93	24.98	0.13
Cl only	27.33	65.73	6.77	0.17
I/Br ratio: 0.5	23.64	0.18	73.40	2.78
I/Br ratio: 1	24.54	0.24	65.10	10.12
I/Br ratio: 2	24.13	0.23	51.29	24.35
I only	23.45	0.17	21.69	54.69

Table S2. The atomic raios of n- and t-PeQD-Br corresponding to Figure 2a . The atomic ratios were measured by XPS.

	Cs [%]	Pb [%]	Br [%]	C [%]	N [%]
n-PeQD-Br	3.91	4.44	11.72	78.77	1.16
t-PeQD-Br	6.37	7.18	23.15	55.34	7.96

Calculation based on atomic ratio to demonstrate ligand exchange:

The calculation process was as follows: first, we assumed that all of oleic acid (OA) and oleylamine (OAM) ligands were changed with acetate and butylammonium (BA) ligands for simplicity. As mentioned in our manuscript, the OA and OAM ligands have eighteen carbons per molecule, whereas the replaced short chain acetate and BA ligands have two and four carbons per molecule, respectively. Additionally, we noted that C/Cs ration changed from 20.14 to 8.68 from the X-ray photoelectron spectroscopy (XPS) data (Figure 2 and Table S2). When we set the ratio of ligands as **a** for OA, **b** for OAM, **c** for acetate, and **d** for BA, we can obtain the following two Equation S3 and S4 by using the C/Cs ratio of n- and t-PeQD-Br films.

$$18\mathbf{a} + 18\mathbf{b} = 20.14\mathbf{k} \quad (3S)$$

$$2\mathbf{c} + 4\mathbf{d} = 8.68\mathbf{k} \quad (4S)$$

In these equations, the **k** was a proportional constant.

Additionally, The N/Cs ratio was increased from 0.30 to 1.25, indicating that the BA ligands passivated the PeQD-Br more densely than OAM after the all-in-one process because only the OAM and BA ligands have one nitrogen atoms in each of the ammonium functional groups (Figure 2a and Table S2). Therefore, we obtained two Equation S5 and S6 signifying the OAM and BA ligands by the N/Cs ratio of n- and t-PeQD-Br.

$$\mathbf{b} = 0.3\mathbf{k} \quad (S5)$$

$$\mathbf{d} = 1.25\mathbf{k} \quad (S6)$$

Through the Equation S5 and S6, we could deduce that the BA ligands passivated the PeQD-Br surface more than OAM ligands approximately 420%. Moreover, by solving the Equation S3, S4, S5, and S6, it was found that the acetate ligands passivated the PeQD-Br surface more than OA ligands by approximately 220%.

References

[1] Q. A. Akkerman, V. D'Innocenzo, S. Accornero, A. Scarpellini, A. Petrozza, M. Prato, L. Manna, *J. Am. Chem. Soc.* **2015**, 137, 10276.

[2] G. P. Li, J. S. Huang, Y. Q. Li, J. X. Tang, Y. Jiang, *Nano Res.* **2019**, 12, 109.

[3]Y. S. Shin, Y, T. J. Yoon, K. T. Lee, J. Jeong, S. Y. Park, G.-H. Kim, J. Y. Kim, *ACS Appl. Mater. Interfaces* **2019**, 11, 23401.

[4]blueY. S. Shin, Y. J. Yoon, K. T. Lee, W. Lee, H. S. Kim, J. W. Kim, H. Jang, M. Kim, D. S. Kim, G.-H. Kim, J. Y. Kim, *ACS Appl. Mater. Interfaces* **2020**, 12, 31582.

[5] W. Jiang, Y. Choi, H. Chae, *J. Mater. Sci.: Mater. Electron.* **2021**, 32, 4686.

[6] a) C. Kim, S.-W. Baek, J. Kim, B. Kim, C. Lee, J. Y. Park, J.-Y. Lee, *ACS Appl. Mater. Interfaces* **2020**, 12, 57840; b) B. Kim, S.-W. Baek, C.Kim, J.Kim, J.-Y. Lee. *Adv. Energy Mater.* **2022**, 12, 2102689.

[7] Y. Hassan, J. H. Park, M. L. Crawford, A. Sadhanala, J. Lee, J. C. Sadighian, E. Mosconi, R. Shivanna, E. Radicchi, M. Jeong, C. Yang, H. Choi, S. H. Park, M. H. Song, F. De Angelis, C. Y. Wong, R. H. Friend, B. R. Lee, H. J. Snaith, *Nature* **2021**, 591, 72.

[8] L. Zhou, K. Yu, F. Yang, H. Chon, N. Wang, J. Zheng, Y. Zuo, C. Li, B. Cheng, Q. Wang, *J. Mater. Chem. C* **2017**, 5, 6224.

[9] H. Chen, Y. Wang, J. Wang, W. Liu, *Coatings* **2021**, 11, 953.

[10] S. Ruan, M.-A. Surmiak, Y. Ruan, D. P. McMeekin, H. Ebendorff-Heidepriem, Y.-B. Cheng, J. Lu, C. R. McNeill, *J. Mater. Chem. C* **2019**, 7, 9326.
